# Endometrial Serous Carcinoma Arising From Adenomyosis

**DOI:** 10.7759/cureus.109441

**Published:** 2026-05-22

**Authors:** Karo K Arzoo, Maurice J Berkowitz

**Affiliations:** 1 Hematology and Medical Oncology, University of California, Los Angeles, Los Angeles, USA

**Keywords:** endometrial serous carcinoma, gynecology-oncology, malignant uterine cancer, rare uterine cancer, uterine cancer

## Abstract

Endometrial serous carcinoma (ESC) is a high-grade and aggressive form of endometrial cancer. Its development in association with adenomyosis, a benign condition characterized by the presence of endometrial tissue within the myometrium, is rare and poorly understood. This case report discusses a postmenopausal woman diagnosed with uterine serous carcinoma arising from adenomyosis, highlighting diagnostic challenges, clinical management, and prognosis. A comprehensive review of the literature is included to offer a broader perspective on this unusual association.

## Introduction

Endometrial serous carcinoma (ESC) is an uncommon high-grade subtype of endometrial cancer. It accounts for 10% of endometrial cancers and is responsible for more than 50% of endometrial cancer-related deaths. It is associated with aggressive clinical behavior and a disproportionately high risk of disease-related mortality, with a five-year overall survival rate of approximately 28.5%. In contrast to endometrioid endometrial carcinoma, ESC is typically estrogen-independent and does not require the presence of estrogen signaling to proliferate. It frequently arises in an atrophic endometrium and demonstrates early myometrium invasion. It is also associated with a propensity for extrauterine dissemination, even in cases with limited uterine disease [1].

Adenomyosis is a common and benign condition defined by the presence of endometrial gland and stroma within the myometrium. Although malignant transformation arising within adenomyosis has been reported, such cases are rare and most frequently involve endometrioid adenocarcinoma. Reports of non-endometrioid malignancies arising from adenomyosis are exceedingly uncommon, with only 44 documented cases. The true incidence is unknown [2].

Serous carcinoma arising from adenomyosis presents a diagnostic challenge, particularly in the absence of a dominant endometrial lesion. Approximately 23% of uterine serous carcinomas present without a visible endometrial lesion on ultrasound, and approximately 37% have extrauterine disease at diagnosis despite no evidence of endometrial stromal or myometrial invasion [3]. This case report discusses a postmenopausal woman diagnosed with uterine serous carcinoma arising from adenomyosis, highlighting diagnostic challenges, clinical management, and prognosis.

## Case presentation

A 55-year-old postmenopausal woman with a history of what appears to be a cryptogenic cirrhosis presents with abnormal vaginal bleeding, pelvic pain, and abdominal bloating for the past 2-3 months. The patient had no known family history of endometrial or ovarian cancer. On physical examination, the uterus was enlarged, measuring 10 cm in length, with an estimated volume of 300 mL. This corresponds to a 12-week gravid uterus. There was no palpable adnexal mass. The abdomen was distended with evidence of shifting dullness.

Ultrasound revealed an enlarged uterus with a heterogeneous myometrial texture, indicative of adenomyosis, and a thickened endometrium (Figure 1). Abdominal MRI revealed splenomegaly (16 cm in length), mild-to-moderate ascites, and a posterior fundal 5 cm uterine fibroid involving the posterior uterine corpus with extension to the cul-de-sac and the involvement of the pericolic tissue (Figure 2).

**Figure 1:**
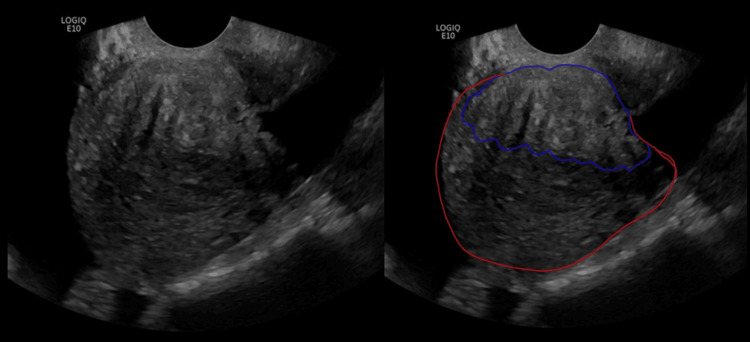
Ultrasound. The red line shows the margin of the enlarged uterus. The blue line encircles the heterogeneous myometrial texture that is indicative of adenomyosis.

**Figure 2:**
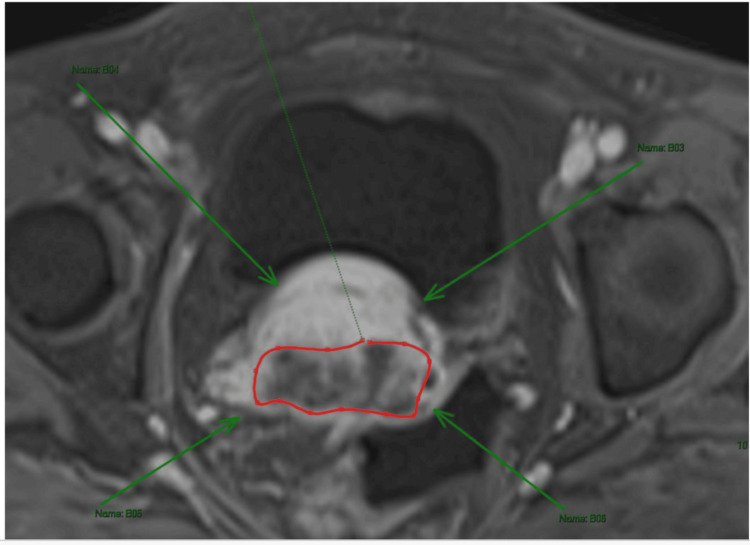
MRI of the pelvis. Posterior uterine fundal mass (circled in red) with extension into the cul-de-sac.

Diagnostic paracentesis was performed and was nondiagnostic. The patient's case was then discussed at the multidisciplinary board. The images were reviewed with radiology, and gynecology-oncology opted to proceed with surgical exploration. She ultimately underwent a total abdominal hysterectomy, bilateral salpingo-oophorectomy, sigmoid colectomy, liver biopsy, and pelvic lymph node dissection. The final pathology revealed serous carcinoma, high-grade, extensively involving the uterine corpus with extension to the cul-de-sac. There is an involvement of the pericolic adipose tissue (Figure 3). There was evidence of adenomyosis involved by carcinoma. In addition, the low anterior resection of the sigmoid colon showed carcinoma involving the pericolic adipose tissue. Two out of nine lymph nodes were positive for macrometastasis. The tumor was positive for aberrant p53 expression by immunohistochemistry (IHC) and negative for human epidermal growth factor receptor-2 (HER2) overexpression (IHC 0). The liver biopsy was negative for malignancy, but incidental liver fibrosis and nodules were noted.

**Figure 3:**
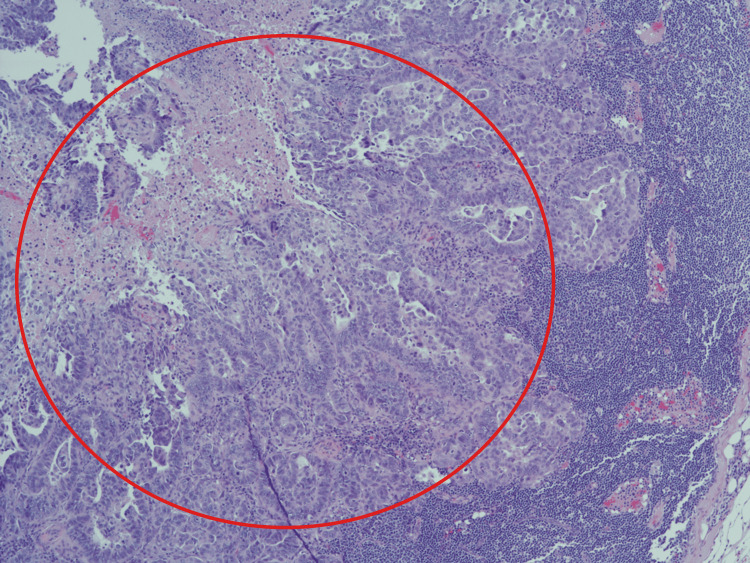
Serous carcinoma, high-grade, extensively involving the uterine corpus with extension to the cul-de-sac. The involvement is noted inside the red circle.

Postoperative staging revealed no distant metastasis, and the tumor was staged as a 2023 International Federation of Gynecology and Obstetrics (FIGO) stage IVB. The patient was subsequently treated with adjuvant chemotherapy (carboplatin and paclitaxel). However, after the first cycle of chemotherapy, she sustained grade 2/3 sensory peripheral neuropathy, diarrhea, and neutropenic fevers. She recovered from the neutropenic fever, but given her deconditioning, she refused further therapy. Two months later, she died, and the cause was attributed to tumor progression.

## Discussion

The incidence of ESC has risen globally and is estimated at 47,400 cases yearly. The increase has been noted in regions where adverse risk factors such as obesity, advanced age, poor lifestyle choices, non-White ethnic groups, and the paucity of medical care are prevalent [2]. A retrospective study conducted in Brazil revealed a median age of 63 years and advanced disease stages at presentation. This study suggested that the main reason for the advanced stage of ESC at identification was diagnostic delays due to limited healthcare access [1]. A second study, conducted in Caribbean-born Black women, pointed toward discrepant clinical outcomes among various racial and ethnic groups. Women in the study seemed to exhibit an increased prevalence of high-grade endometrial cancers, including ESC, when compared to their US-born counterparts. The second study affirms significant differences in stage at diagnosis, poorer prognosis, survival outcomes, and delayed access to healthcare in this underserved population of patients [2].

However, it is important to note that a firm relationship between adenomyosis and ESC still evades the scientific world. The latter may partially be due to the timing and the technical challenges of such a diagnosis. These assumptions are supported by case reports suggesting that serous endometrial intraepithelial carcinoma (SEIC), which can arise in the context of adenomyosis, may often go unrecognized [4]. The diagnostic barrier of adenomyosis in the context of ESC is reflected in current research and clinical trials. Therefore, the research attempting to clarify the role of adenomyosis as a precursor or cofactor in tumorigenesis is currently on a difficult path [5].

The incidence of ESC in relation to adenomyosis has shown varying trends across different countries. These trends are influenced by demographic factors, healthcare access, and lifestyle changes. In Hong Kong, a three-decade-long comprehensive analysis indicated a significant rise in endometrial cancer incidence, with rates escalating from 7.4 to 31.0 per 100,000 people between 1992 and 2020. This trend was mostly seen in women aged 50-65. The study suggested that aging demographics, obesity, and reproductive health behaviors may be causal to the increased incidence of ESC [6]. Similar trends were seen in Taiwan. As such, a retrospective cohort study revealed a rise in the incidence of uterine corpus cancer rates from 5.3 to 15.21 per 100,000 women between 1998 and 2017. Notably, the increase was especially pronounced among women over 50 years of age, indicating a demographic shift that aligns with the aging population [7]. In the United States, the ESC incidence rates follow racial patterns, with non-Hispanic Black women experiencing higher rates of non-endometrioid carcinoma compared to their non-Hispanic White counterparts. This disparity reflects socio-economic factors affecting access to healthcare and the timing of the initial diagnosis [8].

Despite the increasing recognition of these adverse trends, research limitations in the current body of literature continue to persist. Many studies focus on specific populations or regions, somewhat obscuring the global context of ESC. Comprehensive, multicentric studies that account for the above-noted variables are desperately needed to increase our understanding of patient population trends in ESC globally. This is especially urgent as the incidence of early-onset uterine cancers is increasing among all racial and ethnic groups and among younger women, in particular. Studies point to potential links between lifestyle factors, obesity, and the growing incidence of ESC [9].

While uncommon, the association of ESC and adenomyosis has been documented in the literature. The biology of ESC involves a range of genetic, hormonal, and environmental factors that contribute to tumor development and progression. It is crucial to understand the biological basis of this disease to improve both the diagnostic and treatment strategies of patients with ESC [10].

At the molecular level, ESC is often associated with significant genetic alterations, particularly in the *TP53* gene, which is implicated in the regulation of the cell cycle and apoptosis. The *TP53* mutations are commonly observed in most high-grade endometrial cancers and correlate with poor prognosis [11]. The ESC classified as a p53-abnormal type is characterized by high somatic copy number alterations and low mutation burden [11]. Though the molecular stratifications allow for better risk assessment, at this juncture, this is only a fertile ground for research.

The mechanisms linking benign adenomyosis to ESC remain unclear [10]. Prolonged estrogen exposure is known to stimulate the proliferation of endometrial cells and adenomyosis. Epidemiological studies indicate that women with adenomyosis have a higher risk of developing most endometrial cancers. However, the development of ESC is thought to be estrogen-independent [11]. Further research is needed to elucidate the pathogenesis of ESC [12].

ESC is characterized by distinct genomic alterations, which are associated with poor prognosis and advanced disease stage at diagnosis. The presence of these mutations and others underlines the importance of genetic profiling to guide treatment and clinical trial options [13]. The next-generation sequencing (NGS) tools in cervicovaginal samples continue to provide clinicians with insight into the potentially actionable mutations to be used in clinics today [13].

The biology of ESC in the context of adenomyosis is particularly notable. Endometrial tissue within the myometrium can lead to chronic inflammation and hormonal imbalances, thus leading to malignant transformation. This relationship is further complicated by the presence of low allelic frequency mutations in endometrial polyps, which are often coexisting lesions in patients with adenomyosis. In adenomyotic tissue, the accumulation of these mutations may be enhanced by the lack of the natural cyclical shedding seen in normal regenerating endometrial tissue. These mutations correspond to known cancer drivers and suggest a potential precursor role for such benign lesions on the route to their malignant counterparts [14].

Despite ongoing research, there are many limitations in the current genomic understanding of ESC arising from adenomyosis. This is because many studies focus on specific populations or utilize limited sample sizes. Hence, these studies may not be representative of the broader demographics that would encompass a wider genetic diversity. In summary, the relationship between adenomyosis and the specific genomic alterations that lead to ESC requires further investigation [15,16].

Genetic and microenvironmental factors drive the pathophysiology of ESC arising from adenomyosis. ESC is recognized as a particularly aggressive subtype of endometrial cancer, accounting for a disproportionate share of cancer-specific mortality rates despite its low incidence. One of the defining characteristics of this carcinoma is the frequent mutation of the *TP53* gene that regulates the cell cycle and apoptosis. Alterations of this gene can lead to uncontrolled cell proliferation and tumor progression [17,18]. In addition, the presence of ectopic endometrial tissue within the myometrium can lead to a local inflammatory reaction promoting genetic instability and potentially leading to neoplastic pathology [19,20]. The molecular profiling of such cancers has revealed the presence of extensive DNA copy number alterations and significant genomic instability [21,22]. These findings highlight the importance of genomic analysis in understanding the pathophysiology of adenomyosis as it relates to ESC. This is because traditional histopathological parameters do not fully capture the complexity of ESC tumorigenesis. Therefore, we need studies that are committed to the discovery of biomarkers or therapeutic targets to be used in future clinical practice [23,24].

Patients typically present with abnormal uterine bleeding and pelvic pain. These symptoms are common in both adenomyosis and endometrial cancer. As such, the recognition of the underlying ESC may be confounded or delayed [19]. Furthermore, the histopathological characteristics of ESC, particularly in the setting of adenomyosis, also influence clinical presentation. Studies suggest that adenomyosis is commonly found in patients with endometrioid-type endometrial adenocarcinoma, which can coexist with and/or progress to serous carcinoma [19,20].

The overlapping symptoms of adenomyosis and endometrial cancer can lead to misdiagnosis or delays in appropriate treatment. There is a dire need among healthcare providers for an increased awareness of ESC in patients with adenomyosis [19,24]. Unfortunately, a lack of consensus on the pathological criteria that differentiate ESC arising from adenomyosis versus other endometrial cancers further impedes the correct diagnostics and clinical decision-making in this setting [19].

The prognosis for ESC arising is worse compared to other endometrial cancer subtypes. The pathophysiology is driven by high-grade tumors, the presence of *TP53*, extensive myometrial and nodal involvement, and the presence of metastatic disease at diagnosis [18,21]. Delays in diagnosis, chronic inflammation associated with adenomyosis favoring tumor progression, the presence of phosphatidylinositol 3-kinase/protein kinase B (PI3K/AKT) pathway alterations, and high levels of genomic instability (associated with treatment resistance) all contribute to the dismal five-year survival rate of less than 30% in advanced ESC [25-28].

Patients with adenomyosis may face additional challenges in prognosis due to the overlapping symptoms. The presence of adenomyosis can lead to abnormal uterine bleeding and pelvic pain, which may obscure the signs of malignancy, thus delaying timely interventions [27]. Additionally, the chronic inflammation associated with adenomyosis may contribute to a microenvironment that favors tumor progression, adding another layer of adversity to the prognosis [28].

Current treatments for ESC arising from adenomyosis include surgery, chemotherapy, radiation, and possibly emerging targeted therapies. Effective treatment for ESC, especially given its aggressive nature, includes a total abdominal hysterectomy with bilateral salpingo-oophorectomy for localized disease [18]. However, given the propensity of ESC for early metastasis, the surgical approaches are often followed by adjuvant treatments.

Chemotherapy plays a crucial role in the management of advanced ESC. Standard regimens often involve combinations of carboplatin and paclitaxel. In all comers, carboplatin with paclitaxel yields a median progression-free survival improvement of 15 months and a median overall survival of 29 months [17]. Specific molecular aberrations in ESC, such as mutations in the *TP53* gene and alterations in the PI3K/AKT pathway, are associated with poor treatment responses and worse outcomes [18].

Emerging targeted therapies and immunotherapies also hold promise for treating ESC. Investigational agents that target specific molecular pathways involved in tumor progression are being explored in clinical trials. For instance, inhibitors of the PI3K/AKT/mammalian target of rapamycin (mTOR) pathway are under investigation, given the frequent activation of this pathway in ESC [18]. Additionally, immune checkpoint inhibitors, which have shown success in various malignancies, are being evaluated for their potential role in ESC, especially in patients with specific molecular markers indicating heightened immunogenicity and increased tumor mutation burden (TMB). It is important to note that all modern and emerging therapeutics are yet to have relevance in advanced ESC. Therefore, the need for ongoing research in this space could not be overemphasized [11,26].

Clinical trials are increasingly focusing on the molecular characteristics of endometrial cancers, which have significant implications for treatment strategies. The Cancer Genome Atlas (TCGA) has classified endometrial carcinoma into distinct molecular subtypes, enabling more personalized approaches to therapy, particularly for aggressive forms such as ESC [28,29]. Current trials aim to assess the efficacy of targeted therapies that address specific genomic alterations, such as those in the PI3K/AKT pathway and HER2/neu, which are seen in ESC [30,31].

One notable trial examines the role of serous endometrial intraepithelial carcinoma (SEIC), a precursor to ESC. In this trial, the researchers are evaluating the safety and efficacy of simpler surgical interventions in patients [32,33]. This study seeks to determine whether less invasive surgical options can be safely applied without compromising patient outcomes, thus potentially reshaping surgical management protocols for early-stage ESC. Additionally, trials are examining the relationship between surgical approaches and molecular characteristics to optimize treatment strategies tailored to individual patients' tumor profiles [34]. Such investigations are critical as they could enhance the precision of surgical selections and improve long-term outcomes for patients with ESC, particularly those arising from adenomyosis.

Despite these advancements, several challenges persist within the clinical trial landscape. The rarity of ESC and its association with adenomyosis complicate recruitment for large-scale trials, which often limits the generalizability of findings [30,32]. Additionally, many studies to date have primarily focused on endometrioid adenocarcinoma, with relatively few dedicated to serous carcinoma, underscoring the need for more comprehensive research on this specific subtype [34,35]. Furthermore, the diagnostic complexities associated with differentiating adenomyosis from malignancy often lead to delays in treatment initiation, further complicating patient trial recruitment [36,37].

These are some of the examples of drug candidates that are being studied as treatment for ESC. Elacestrant is another potential candidate for the treatment of ESC. It is a drug targeting estrogen receptor (ER)-positive malignancy and is primarily indicated for breast cancer. However, it could be potentially used in ER-positive ESC [38]. Cytalux (pafolacianine) is an optical imaging agent specifically indicated for ovarian cancer but could potentially be used for intraoperative applications in endometrial cancer surgeries. Cytalux could enable more complete resections of ESC arising from adenomyosis by aiding in the identification of malignant lesions [39]. Acr-368 is an investigational drug that targets specific kinases involved in tumor cell proliferation and survival. This creates an opportunity to treat advanced solid tumors, including endometrial cancers. Acr-368 focuses on disrupting signaling pathways critical to tumor growth, thereby offering a potential new treatment avenue for resistant forms of ESC [40]. Ogsiveo is currently indicated for progressing desmoid tumors but demonstrates a movement toward diverse treatment strategies within oncology. Although it is not directly indicated for endometrial serous carcinoma, its systemic treatment approach may inspire new therapeutic modalities that could be adapted for use in cases of adenomyosis-related malignancies [41]. Anktiva, which is utilized in combination with Bacillus Calmette-Guérin (BCG) for bladder cancer, represents a growing trend in immunotherapeutic strategies. This drug has no indications for endometrial cancer. However, the immunological principles could inspire research into similar approaches for treating endometrial serous carcinoma, particularly in patients with adenomyosis, where immune modulation may play a role in tumor dynamics [40].

The ongoing research and clinical trials for these various drugs highlight a varied approach to treating endometrial serous carcinoma arising from adenomyosis. Trends indicate a shift toward personalized medicine, leveraging molecular and genetic understanding to inform therapeutic strategies. However, limitations persist, particularly regarding the rarity of this specific cancer subtype, which may hinder the breadth of clinical data available for these drugs. As more studies are conducted, the clinical community will gain a deeper understanding of how these interventions can be effectively integrated into treatment paradigms for patients suffering from this rare malignancy.

## Conclusions

Despite modern cancer therapeutic advancements, many limitations exist in the management landscape for ESC arising from adenomyosis. The rarity of this disease and the overlapping symptoms of adenomyosis with those of ESC lead to delays in diagnosis and the timing of treatment initiation, ultimately affecting patients adversely. Continued research is essential to better understand the histopathological, psychosocial, and molecular basis of ESC. Large-scale trials are needed to pave the way for future treatments that would enhance the survival and quality of these patients.

## References

[1] Anton C, Kleine RT, Mayerhoff E (2020). Ten years of experience with endometrial cancer treatment in a single Brazilian institution: patient characteristics and outcomes. PLoS One.

[2] Schlumbrecht M, Huang M, Hurley J, George S (2019). Endometrial cancer outcomes among non-Hispanic US born and Caribbean born black women. Int J Gynecol Cancer.

[3] Kiff JM, Williams-Weisenberger M, Spellacy D, Garg B, Munro EG, Bruegl AS (2023). Ultrasonographic evaluation of endometrial stripe thickness is insufficient to rule out uterine serous carcinoma. Cancer Causes Control.

[4] Abushahin N, Zhang T, Chiang S, Zhang X, Hatch K, Zheng W (2011). Serous endometrial intraepithelial carcinoma arising in adenomyosis: a report of 5 cases. Int J Gynecol Pathol.

[5] Essmat AA (2021). Study of prevalence of endometrial cancer in patients with adenomyosis uteri. Int J Reprod Contracept Obstet Gynecol.

[6] Ma X, Wu X, Du J, Sun H (2024). Evolution of endometrial cancer incidence patterns in Hong Kong: a three-decade analysis with future projections. Heliyon.

[7] Tai YJ, Chiang CJ, Chiang YC, Wu CY, Lee WC, Cheng WF (2023). Age-specific trend and birth cohort effect on different histologic types of uterine corpus cancers. Sci Rep.

[8] Clarke MA, Devesa SS, Harvey SV, Wentzensen N (2019). Hysterectomy-corrected uterine corpus cancer incidence trends and differences in relative survival reveal racial disparities and rising rates of nonendometrioid cancers. J Clin Oncol.

[9] Wijayabahu AT, Clarke MA (2024). Abstract B021: early onset uterine corpus cancer incidence rates and 5-year relative survival by histologic subtype and race/ethnicity among women aged 20-49 years. Clin Cancer Res.

[10] Oaknin A, Bosse TJ, Creutzberg CL (2022). Endometrial cancer: ESMO Clinical Practice Guideline for diagnosis, treatment and follow-up. Ann Oncol.

[11] Katagiri R, Iwasaki M, Abe SK (2023). Reproductive factors and endometrial cancer risk among women. JAMA Netw Open.

[12] Gao Y, Wang H, Jiang M, Cui Y, Yu X (2025). Fertility-sparing treatment for patients with endometrial cancer: a bibliometric analysis from 2000 to 2024. Front Oncol.

[13] Pelegrina B, Paytubi S, Marin F (2023). Evaluation of somatic mutations in cervicovaginal samples as a non-invasive method for the detection and molecular classification of endometrial cancer. EBioMedicine.

[14] Sahoo SS, Aguilar M, Xu Y (2022). Endometrial polyps are non-neoplastic but harbor epithelial mutations in endometrial cancer drivers at low allelic frequencies. Mod Pathol.

[15] Gu J, Zhang H, Qian K, Ye X, Wu G (2024). Who is focusing on women’s health: a bibliometric analysis of global research trends on overweight in female patients with gynecologic or breast cancer. Int J Womens Health.

[16] Al-Mayoofee SH, Missaoui N, Hmissa S, Al-Snafi AE (2024). Adipokines in relation to weight, lipid profile and glycemic state in women with polycystic ovary syndrome. J Obstet Gynecol Cancer Res.

[17] Jayraj AS, Abdul-Aziz S, Mburu A, Upadhyay A, Singh N, Ghatage P (2024). Narrative review on the evolving role of HER2/neu targeting in uterine serous cancers. Ann Transl Med.

[18] Kuhn E, Wu RC, Guan B (2012). Identification of molecular pathway aberrations in uterine serous carcinoma by genome-wide analyses. J Natl Cancer Inst.

[19] Sun Y, Lin S, Wu W (2024). Whether surgical procedure can improve the prognosis of endometrial cancer arising in adenomyosis (EC-AIA)? A systematic review and meta-analysis. Int J Surg.

[20] Conlon N, Da Cruz Paula A, Ashley CW (2020). Endometrial carcinomas with a “serous” component in young women are enriched for DNA mismatch repair deficiency, Lynch syndrome, and POLE exonuclease domain mutations. Am J Surg Pathol.

[21] Lax SF (2017). Pathology of endometrial carcinoma. Adv Exp Med Biol.

[22] Levine DA (2024). Abstract IA002: management of newly diagnosed endometrial cancers and molecular classification. Clin Cancer Res.

[23] Brinton LA, Felix AS, McMeekin DS (2013). Etiologic heterogeneity in endometrial cancer: evidence from a Gynecologic Oncology Group trial. Gynecol Oncol.

[24] Wang A, Chen Y, Holloway RW, Pei Z, Yang J, Zhao Y (2020). Clinicopathologic and molecular genetic comparison of primary uterine serous adenocarcinomas from African American and Caucasian women. J Clin Oncol.

[25] Zhang L, Kwan SY, Wong KK, Solaman PT, Lu KH, Mok SC (2020). Pathogenesis and clinical management of uterine serous carcinoma. Cancers (Basel).

[26] Corr B, Cosgrove C, Spinosa D, Guntupalli S (2022). Endometrial cancer: molecular classification and future treatments. BMJ Med.

[27] Santoro M, Macis C, Coada CA (2024). 341 Artificial intelligence as tool for early diagnosis and precision surgery, in endometriosis-related ovarian cancer (ATENA): the interim analysis. Int J Gynecol Cancer.

[28] Pandur E, Pap R, Jánosa G, Horváth A, Sipos K (2023). The role of fractalkine in the regulation of endometrial iron metabolism in iron deficiency. Int J Mol Sci.

[29] Najjar O, Erickson BK, Nickles-Fader AN (2020). Diagnosis and management of uterine serous carcinoma: current strategies and clinical challenges. Expert Opin Orphan Drugs.

[30] Kuhn TM, Dhanani S, Ahmad S (2023). An overview of endometrial cancer with novel therapeutic strategies. Curr Oncol.

[31] Raffone A, Raimondo D, Maletta M (2023). Endometrial cancer arising in adenomyosis (EC-AIA): a systematic review. Cancers (Basel).

[32] Slaager FC, Hofhuis W, Hoogduin KJ, Groenendijk FH, Van Beekhuizen HJ (2025). Serous endometrial intraepithelial carcinoma (SEIC): a prospective registration study. Int J Gynecol Cancer.

[33] Sheikhvatan M, Chaichian S, Moazzami B (2020). A systematic review and bioinformatics study on genes and micro-RNAs involving the transformation of endometriosis into ovarian cancer. Microrna.

[34] Wang J, Wang Q, Wang W, Yang J, Xia J, Wei Y (2023). Endometrioid adenocarcinoma arising in adenomyosis in a patient with pelvic organ prolapse-case report. BMC Womens Health.

[35] Kumar N, Sharma A, Mangla M, Srirambhatla A (2024). Adenomyosis or endometrial carcinoma? Radiological pitfalls in postmenopausal diagnosis: a case report. Egypt J Radiol Nucl Med.

[36] Moraru L, Mitranovici MI, Chiorean DM, Moraru R, Caravia L, Tiron AT, Cotoi OS (2023). Adenomyosis and its possible malignancy: a review of the literature. Diagnostics (Basel).

[37] Perkey C (2023). Elacestrant (Orserdu™). Oncol Times.

[38] Baron E, Patterson R, Tillman R, Wernberg JA, Sharma R (2025). Real‑world application of Cytalux for targeted imaging of occult peritoneal disease in epithelial ovarian cancer. Mol Clin Oncol.

[39] (2025). A study of ACR-368 in ovarian carcinoma, endometrial adenocarcinoma, and urothelial carcinoma. https://www.cancer.gov/research/participate/clinical-trials-search/v?id=NCI-2022-10629.

[40] OGSIVEO- Nirogacestat Tablet, Film Coated (2026). Label: OGSIVEO- nirogacestat tablet, film coated. DailyMed, U.S. National Library of Medicine, 1 Apr.

[41] Keam SJ (2024). Nogapendekin alfa inbakicept: first approval. Drugs.

